# Development and psychometric evaluation of nurses’ health-related procrastination scale

**DOI:** 10.1016/j.heliyon.2023.e18145

**Published:** 2023-07-10

**Authors:** Mahdi Basirimoghadam, Forough Rafii, Abbas Ebadi

**Affiliations:** a Nursing and Midwifery Care Research Center, Iran University of Medical Sciences, Tehran, Iran; b Behavioral Sciences Research Center, Life Style Institute, Baqiyatallah University of Medical Sciences, Tehran, Iran

**Keywords:** Psychometrics, Health, Nurses, Procrastination

## Abstract

There is no valid scale to measure health related procrastination, especially in nurses. This study was conducted to design and assess the psychometric properties of the health-related procrastination scale in nurses. This exploratory mixed method study was conducted between April 2017 and May 2019. The design and psychometric assessment of the nurses' health-related procrastination scale (NHRPS) was carried out through quantitative and qualitative phases. In the qualitative phase, nurses were interviewed and a review of literature was carried out to generate the items. In the quantitative phase, the scale validity was evaluated using the face, content and construct validity and its reliability was evaluated through the Cronbach's alpha, McDonald's omega, intra-class correlation coefficient, standard error of measurement, minimal detectable changes. The results of the exploratory factor analysis showed that, with 29 items and five factors, the NHRPS explains 54.81% of the variance in NHRPS. The results of confirmatory factor analysis also confirmed the final fit of model with five factors (RAMSEA: 0.08, CMIN/DF: 2.42, NFI: 0.92, PNFI: 0.83, CFI: 0.95, IFI: 0.95, RFI: 0.91, SRMR: 0.074). The Cronbach's alpha of the scale was 0.947, its intra-class correlation coefficient was 0.944, the Half-fold consistency coefficient was 0.970 and Omega internal consistency coefficient was 0.96. The NHRPS has good psychometric properties. This scale is suitable for research purposes, especially to plan for nurses' health promotion.

## 1 Introduction

The physical and mental health of nurses is a determining factor in their occupational efficiency and is associated with the quality of care provided to patients [1]. Of all the factors affecting humans’ health, illness and death, health-related behaviors are a factor that explain the largest variance in premature death [2].

Nurses’ health-related behaviors are astonishing and worrying with respect to their roles in health promotion [3]. Despite general information about the role of behavior in health, unhealthy behaviors are prevalent [4]. The results of a study showed that nurses are more obese and do fewer physical activities than the general population does [3]. Now the question is why awareness of the role of health behaviors does not lead to health behaviors.

Recently, the gap between intention and behavior has attracted considerable attention, and procrastination has been defined as the gap [5]. Procrastination is defined as a voluntary and unnecessary delay in starting or completing tasks despite being aware that the situation is getting worse with a delay [6,7]. Procrastination has negative effects on occupational efficiency, academic performance and psychological well-being [8]. In addition to its negative consequences, procrastination is a common and widespread phenomenon with a prevalence estimated up to 96% among American college students, as about 15–20% of adults suffer from chronic procrastination [9]. It is thus not startling that procrastination has been a focus of research for the past three decades [10].

Procrastination is related to culture [11], so it should be designed and adapted in the target society. Most procrastination scales were designed in the academic setting. Tuckman Procrastination Scale was developed by Tuckman with 72 items in 1991. The results of factor analysis reduced the number of items to 35 with Cronbach's alpha equal to 0.90. In a subsequent study, the results of factor analysis on 35 items reduced the number of items to 16 with Cronbach's alpha of 0.86. Tuckman recommended this shorteneded version to look for procrastination tendencies in completing academic tasks [12].

The Procrastination Assessment Scale for Students is a commonly used measure of academic procrastination that was designed to assess procrastination in two different areas: frequency of procrastination and reasons for procrastination. It is developed by Solomon and Rothblum in 1984. Solomon and Rothblum did not report reliability estimates for the PASS in their original study [13].

Academic Procrastination State Inventory was introduced by Schouwenburg in 1995. It assesses procrastination on a variety of study related activities in academic setting [14]. The General Procrastination Scale was developed by Lay in 1986. This scale examines procrastination in daily tasks such as sporting events, parties, phone calls, and arriving at the airport. This scale was also psychometrically evaluated in the student setting. Cronbach's alpha of the scale was reported as 0.83 [15]. McCown and Johnson (1989) introduced the Adult Inventory of Procrastination to assess general procrastination in adult. This scale focuses on procrastination behaviors in non-academic contexts. Good reliability was reported for this scale, Cronbach's alpha was 0.86 and test-retest reliability was 0.76 [16].

There are several limitations for these scales, they were designed and psychometrically evaluated in an academic setting and there is no scale to measure procrastination in the nursing community and most of these scales measure academic and daily tasks procrastination. However, there is no valid scale to measure HRP, especially in nurses. To the best of the researchers' knowledge, there is only one scale that measures HRP in students [2], and this scale measures mainly procrastination in performing exercises and adhering to diets and does not assess procrastination in other aspects of health such as mental health, spiritual health and social health. Second, some health behaviors, such as washing hands, hepatitis B vaccination, antibody titer control and adherence to safety principles (wearing masks, gowns, gloves and goggles) are particularly crucial for nurses, but not for students and other community members. Third, nurses' information about diseases, their health care responsibilities and also their access to health functions, such as physician visits, differ from students and other members of the community. In general, there is a need for a single scale that measures nurses’ HRP. Accordingly, the present study was conducted to design and validate a scale for HRP measurement in nurses.

## 2 Materials and methods

### 2.1 Design and setting

This study is part of a larger mixed-method research. It was a methodological research which conducted on nurses in teaching hospitals affiliated to Iran University of Medical Sciences (IUMS) and Gonabad University of Medical Sciences (GUMS)- Iran, between April 2017 and May 2019.

The design and psychometric assessment of the scale were carried out through qualitative and quantitative phases. In the qualitative phase of the study, the content analysis approach was used to design and generate items for the scale. In the quantitative phase, the psychometric properties of the scale were assessed.

### 2.2 Qualitative phase

This phase lasted from April to November 2017. First, to design the scale and to generate its items, the concept of HRP and its dimensions were reviewed and explained. The data were collected using purposive sampling with maximum variation in terms of place of service (city and capital), age, gender, work experience, and service area. In this phase, 17 nurses were interviewed. Each interview lasted 40–80 min. The interviews were semi-structured. The main questions that were asked to the participants: “What do you do when you are faced with a problem in your physical condition?” And “Describe one of your health-related procrastinations”. Then, according to the statements of the participants, more detailed information about the concept of the research subject was asked using in-depth questions. Based on the analysis of each interview, the next interview was conducted.

All the interviews were held by the researcher in Persian and then immediately transcribed and translated into English. Data were analyzed according to Graneheim and Lundman's method of content analysis. Immediately after each interview, the interview transcripts were reviewed several times to gain an overall understanding of their content. Meaning units were selected and the primary codes were extracted; the codes were then classified into themes and subthemes based on their similarities and differences. Data were analyzed using MAXQDA 11 software.

#### 2.2.1 Trustworthiness

To increase the credibility of the study, nurses were nominated from a diverse background. Efforts were also made to select the best meanings units, categories, and themes, and the most useful quotations were also drawn from the transcribed interviews. Also member checks increased credibility. The researchers had years of experience working as a nurse and qualitative researchers. The dependability of the study was ensured through external checks and audit. To boost the data confirmability, an audit trail was written and kept; in this way, all the decisions and the stages of the study were documented and their details were fully reported. Finally, transferability was enhanced by doing a thorough job of describing the research context and the assumptions that were central to the research [17].

#### 2.2.2 Item generation

The initial item pool was created based on the results of interviews with nurses and a review of literature. An extensive review of literature was carried out on procrastination and health. Since there were very few studies on HRP, especially in nurses, the studies, books, tools and questionnaires related to the general terms ‘procrastination’ and ‘health’ were reviewed (the keyword ‘nurse’ was eliminated from the search). The search was carried out in Pubmed, ISI Web of science, Scopus, ProQuest, and SienceDirect databases. The initial items of the HRP scale were compiled at the end of this phase.

### 2.3 Psychometric properties

This phase lasted from December 2017 to May 2019. The scale validity was evaluated through face validity, content validity and construct validity and its reliability was evaluated through internal consistency, relative and absolute reliability.

#### 2.3.1 Face validity

Both qualitative and quantitative measures were used to investigate the face validity of the scale. For the qualitative face validity assessment, the researchers held ten interviews with nurses about the difficulty level, relevance and ambiguity of the words, phrases and sentences of the scale. Then, any ambiguous items or sources of misunderstanding were clarified based on the nurses’ opinions.

The quantitative face validity assessment consisted of calculating the item impact scores. A questionnaire was therefore distributed among ten clinical nurses to answer each item on a 5-point Likert scale of importance, from ‘not important at all’ to ‘absolutely important’. Any item with an impact score ≥1.5 was maintained for the subsequent analysis [18]. In order to ensure the correct phrasing and sentence structure of the questionnaire, the statements were reviewed by two Persian literature experts.

#### 2.3.2 Content validity

The content validity was examined using the comments of ten experts in scale development, nursing, occupational medicine, occupational health engineering, community health nursing, psychiatric nursing and medical education. The content validity was examined using the content validity ratio (CVR) and content validity index (CVI). To determine the CVR, ten experts were asked to review each sentence on a 3-point scale (necessary, useful but not necessary, and not necessary), and based on Lawshe's table, the items with CVR ≥0.62 were retained [19]. In order to determine the CVI, ten experts were asked to examine the items' adequacy and to respond to each of them according to the relevance criterion on a 4-point Likert scale. The I-CVI was then calculated for each item and the S-CVI also for the entire scale. The items with a I-CVI score of 0.78 or higher were considered appropriate and kept in the scale. To calculate the S-CVI, the average of the computed I-CVI values was used for each item. Polit and Beck have recommended a score of ≥0.90 for accepting items [19].

#### 2.3.3 Item analysis

Before carrying out the construct validity, the item analysis was done on 50 clinical nurses and the correlation between the items and the correlation of each item with the total score of the instrument were calculated. If the correlation coefficient between the item and the entire instrument was less than 0.3, the item was deleted. Also, if the correlation coefficient between two items was more than 0.7, one of the items was deleted. At this phase, the Loop method was used for item analysis. In the loop method, the reliability coefficient of all questions is calculated first. If the reliability level is reduced by eliminating the questions, it indicates that this question plays an effective role and is in coordination with other questions, and therefore the question is appropriate.

#### 2.3.4 Construct validity

##### 2.3.4.1 Exploratory factor analysis

The Maximum Likelihood Estimation was first used for the factor analysis of the nurses’ health related procrastination (NHRPS). A varimax rotation was then applied to identify the fundamental factors underlying the scale and to achieve a simple structure. Factor extraction was performed after calculating the correlation matrix between the items. In the factor analysis, the criterion was set as factor loadings more than 0.4 [20]. Eigenvalues and Scree plots were used to determine the number of effective factors in the scale [21]. Exploratory factor analysis was performed using SPSS 14.5 (SPSS Inc., Chicago, IL, USA).

##### 2.3.4.2 Confirmatory factor analysis

In confirmatory factor analysis, the most common goodness of fit indicators of the proposed model was evaluated based on the threshold of acceptance in maximum likelihood estimation using the confirmatory factor analysis. Therefore, the normed fit index (NFI), non-normed fit index (NNFI), parsimony normed fit index (PNFI), goodness of fit index (GFI), adjusted goodness of fit index (AGFI), incremental fit index (IFI), relative fit index (RFI), comparative of fit index (CFI), root mean square error of approximation (RMSEA) and Chi-Square were investigated. Confirmatory factor analysis was performed using LISREL version 8.8.

##### 2.3.4.3 Sample size and participants

There are different views on sample size in exploratory factor analysis, but usually, the number of samples should be five to ten times as much as the number of items [22]. In the present study, nine samples were taken for each item (315 nurses). The Kaiser-Meyer-Olkin (KMO) test and Bartlett's test were used to determine the adequacy of the samples and also the adequacy of the factor analysis for the data. Given that the minimum sample size required to perform confirmatory factor analysis is 200, we selected 222 participants. Convenience sampling was used to select the participants from hospitals affiliated with IUMS and GUMS.

#### 2.3.5 Reliability

The scale reliability was determined by internal consistency, relative and absolute reliability. Cronbach's alpha, Omega internal consistency coefficient and Half-fold consistency coefficient were measured to determine the internal consistency. The relative reliability of the scale was examined through its completion by 50 nurses at two-week intervals and by obtaining an intra-class correlation (ICC) for the items. Absolute reliability was calculated by standard error of measurement and minimal detectable changes. Data were analyzed in SPSS 14.5 (SPSS Inc., Chicago, IL, USA).

### 2.4 Ethical considerations

An ethical approval was obtained from the Ethics Committee of IUMS (IR.IUMS.REC 1395.95-03-123-29560) to carry out this research and all the participants submitted informed consent forms prior to participation.

## 3 Results

Table 1 shows the nurses demographic characteristics for exploratory and confirmatory factor analysis. The mean of the nurses' age for exploratory and confirmatory factor analysis was 31.50 ± 7.35 and 30.55 ± 8.15 years, respectively (Table 1).

**Table 1:** Demographic characteristic of the nurses.

Variable		Exploratory Factor Analysis	Confirmatory Factor Analysis
**Age**	Mean (SD)	31.50 (7.35)	30.55 (8.15)
**Work Experiences**	Mean (SD)	8.27 (7.21)	9.31 (7.88)
**Residence**			
Gonabad	N (%)	150 (50.0)	122 (55.0)
Tehran	N (%)	150 (50.0)	100 (45.0)
**Gender**			
Male	N (%)	82 (27.3)	78 (36.3)
Female	N (%)	218 (72.7)	137 (63.7)
**Marital status**			
Single	N (%)	80 (26.7)	73 (33.2)
Married	N (%)	218 (72.7)	147 (66.8)
Widow	N (%)	1 (0.3)	0 (0.0)
divorced	N (%)	1 (0.3)	0 (0.0)
**Education**			
Bachelor's	N (%)	201 (67.0)	166 (75.5)
Master's	N (%)	97 (32.3)	54 (24.5)
PhD	N (%)	2 (0.7)	0 (0.0)
**Shift**			
In circulation	N (%)	222 (74.0)	162 (74.7)
Fixed	N (%)	78 (26.0)	55 (25.3)

In the qualitative phase, the initial items of the NHRPS were extracted and the initial item pool was created with 80 items. Subsequently, the items developed by the research team were reviewed and rewritten and their comments were summarized and duplicate or similar items were removed. The items agreed upon by all or most of the research team were selected. At the end of this phase, 49 items remained in the scale.

During the face validity assessment phase, two items were removed because their impact score was less than 1.5. In the content validity assessment, four items were eliminated because they had a CVR <0.62. In the CVI assessment phase, four items were deleted because they had a CVI <0.78. The S-CVI for the entire scale was obtained as 94.9. The kappa coefficient for all of the items was above 0.74 that meant an excellent level.

In the item analysis phase, the total Cronbach's alpha value for this instrument was 0.95. In the column, Cronbach's Alpha if item deleted, all items had a value above 0.94. At this phase, one item was deleted due to a correlation of less than 0.3 with the total score of the other items and three items were removed or merged due to a correlation of above 0.7 with another item, and in total, at this phase, four items were removed or merged.

**Fig. 1:**
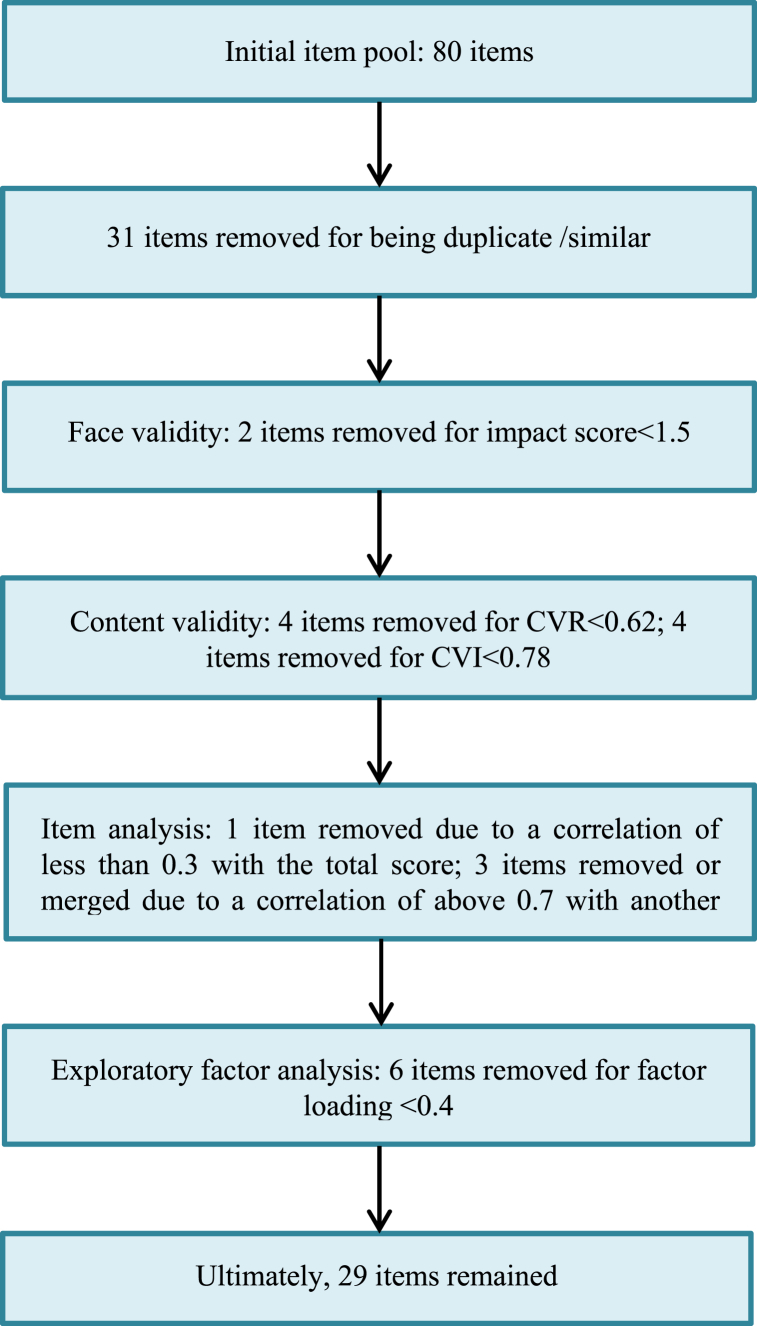
A summary of the development of the NHRPS

Thirty-five items remained in the scale for the exploratory factor analysis step, in which six items were removed from the questionnaire for failure to reach the minimum factor loading. Ultimately, 29 items remained in the scale (Fig. 1).

The KMO test was 0.916 in this study, which indicates that the data were sufficient for the analysis. Bartlett's test was also significant (χ2300 = 5416.03, p < 0.001), which indicates a sufficient correlation between the variables for performing factor analysis. These two tests demonstrated that the items were sufficient for performing factor analysis.

Based on the results of the exploratory factor analysis, seven factors were initially extracted with Eigenvalues greater than 1 and based on the Scree plot. After performing the factor analysis for six times and eliminating the items that were not loaded on any factor, the five-factor model was found to have a better fit with regard to item ordering and factor naming (Table 2).

**Table 2:** Nurses’ health related procrastination scale face validity, content validity and exploratory factor analysis.

Factors	Item	IS	CVR	CVI	Kappa	Communalities	Factor loading	%Variance
Procrastination in maintaining physical health	7. I delay my doctor's visit, although the doctor is available	2.22	0.8	1	1	0.829	0.855	15.8
	8. I'm late for tests (i.e. pap smear, X-ray), although appointments are available	3.2	0.8	0.9	0.89	0.811	0.836	
	6. I delay my medical checkups, although medical services are available	4.05	1	1	1	0.677	0.735	
	9. I delay monitoring my blood pressure, although devices are available	2.73	0.8	0.8	0.79	0.510	0.633	
	5. I delay washing my hands, although it is essential for nurses' health	4.6	0.8	1	1	0.393	0.554	
	4. I delay adhering to the principles of ergonomics (proper body positions) when providing nursing care	3.87	0.8	1	1	0.394	0.550	
	3. I delay getting vaccinated and controlling my antibody titers for hepatitis, although these are essential for nurses	4.8	0.8	1	1	0.313	0.489	
	18. I delay adhering to safety principles (gowns, masks, gloves, glasses) when providing nursing care	3.44	0.8	0.9	0.89	0.386	0.467	
Procrastination in physical health promotion	12. I delay weight control program	2.87	1	1	1	0.590	0.728	6.62
	13. I delay having a healthier diet	3.44	1	1	1	0.566	0.577	
	11. I delay exercising	2.73	1	1	1	0.451	0.514	
	10. I delay having enough sleep and rest, although they are necessary for nurses' health	2.8	0.8	1	1	0.355	0.421	
Procrastination in quitting high-risk behaviors	16. I delay quitting substance abuse (i.e. opioid, drugs)	2.16	0.8	1	1	0.966	0.980	7.22
	17. I delay quitting drinking alcohol	1.6	0.8	1	1	0.734	0.850	
	15. I delay quitting tobacco use (i.e. cigarettes, hookah)	2.73	1	1	1	0.298	0.500	
Procrastination in social and mental health	24. I delay meeting my friends and relatives	2.04	0.8	0.9	0.89	0.589	0.702	15.04
	25. I delay socializing with my colleagues	2.34	0.8	0.9	0.89	0.588	0.674	
	31. I delay participating in group activities with my colleagues	2.04	0.8	1	1	0.571	0.635	
	23. I delay releasing my feelings after dealing with stressful cases in the hospital	2.28	0.8	1	1	0.497	0.627	
	22. I delay activities that make me happy (i.e. travel and leisure)	3.44	0.8	0.8	0.79	0.473	0.581	
	26. I delay the establishment of intimate relationships with my family	4.14	0.8	0.8	0.79	0.449	0.568	
	27. I delay managing my finances	1.98	0.8	0.8	0.79	0.519	0.554	
	30. I delay pursuing things that improve my social status (i.e. higher education)	2.16	0.8	1	1	0.443	0.545	
	29. I delay learning new skills and acquiring new knowledge related to my profession	3.36	0.8	0.8	0.79	0.442	0.477	
	21. I delay seeking help and treatment for my mental health problems	2.52	1	1	1	0.360	0.411	
Procrastination in spiritual health	33. I delay attending to my spirituality	2.45	0.8	1	1	0.895	0.902	10.09
	35. I delay my spiritual obligations	3.6	0.8	0.9	0.89	0.629	0.729	
	34. I delay reading spiritual books	1.8	1	1	1	0.542	0.637	
	32. I delay attending spiritual ceremonies	1.98	0.8	0.8	0.79	0.627	0.610	

Note. IS = impact score; CVR = content validity ratio; CVI = content validity index.

The five extracted factors accounted for 54.81% of the commutative variance, and the remaining 45.19% of the total variance was explained by the 24 remaining factors with Eigenvalues less than 1. The scree plot also represents five factors in this exploratory factor analysis.

The first factor obtained from the factor analysis, i.e. procrastination in maintaining physical health, had eight items. The second factor, i.e. procrastination in physical health promotion, had four items. The third factor, i.e. procrastination in quitting high-risk behaviors, had three items. The fourth factor, i.e. procrastination in social and mental health, had ten items. The fifth factor, i.e. procrastination in spiritual health, had four items.

**Fig. 2:**
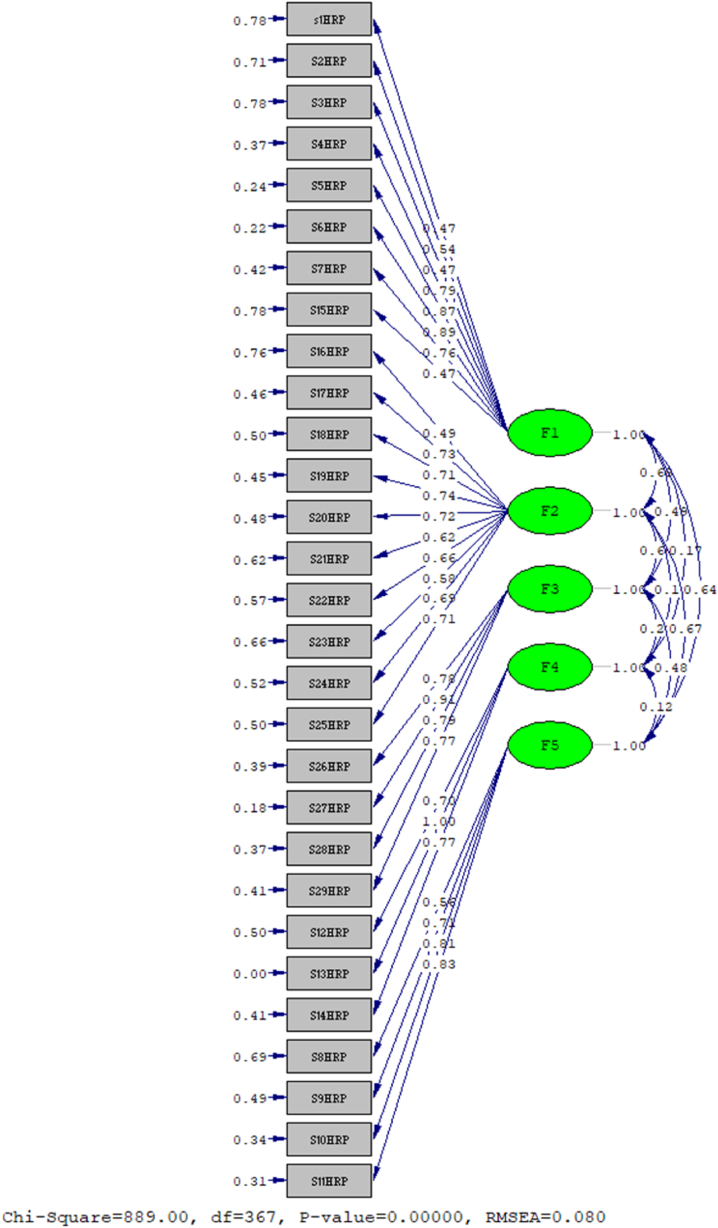
The final structure of the NHRPS model.

The confirmatory factor analysis indicated a reliable estimate of the model with its general fit indices (Table 3). The variables in the NHRP structure had a strong alignment with their appropriate factors, which is seen in Fig. 2(Fig. 2)

**Table 3:** The accepted threshold of indexes and fitting of the confirmatory factor analysis model.

Fitting Indexes	Acceptable Range	Our Results
X2 P-value RMSEA CFI NFI NNFI PNFI SRMR CMIN/DF IFI RFI	>0.05 Good <0.08, medium 0.08 to 0.1, and weak <0.1 >0.9 >0.9 >0.9 >0.5 <0.1 Good<3, Acceptable<5 >0.9 >0.9	0.001 0.08 0.95 0.92 0.94 0.83 0.07 2.42 0.95 0.91

Note. RMSEA = root mean square error of approximation; CFI = comparative fit index; NFI = normed fit index; NNFI = non-normed fit index; PNFI = parsimony normed fit index; SRMR= Standardized root mean square residual; CMIN/DF = discrepancy divided by degree of freedom; IFI = incremental fit index; RFI = relative fit index.

To determine the reliability of the scale, both internal consistency and test-retest stability methods were used in a sample of 50 randomly-selected nurses. The Cronbach's alpha of the entire scale was 0.947. The Cronbach's alpha was 0.891 for the first factor, 0.885 for the second factor, 0.901 for the third factor, 0.890 for the fourth factor, and 0.886 for the fifth factor. Omega internal consistency coefficient of the entire scale was 0.96. The Omega was 0.94 for the first factor, 0.93 for the second factor, 0.91 for the third factor, 0.93 for the fourth factor, and 0.86 for the fifth factor. The half-fold coefficient of the entire scale was 0.970. After dividing the scale into two parts according to odd even numbers, the half-fold reliability was 0.898 for the first part and 0.895 for the second part. The ICC coefficient was above 0.85 for the entire scale and all the factors (Table 4).

**Table 4:** The reliability of the nurses’ health-related procrastination scale using internal consistency, absolute and relative stability.

Factor	Mean (SD)	N	α	Omega	ICC^a^	95%CI	p-value	SEM	MDC	MDC%	Result
Procrastination in maintaining physical health	22.20 (6.88)	8	0.891	0.94	0.923	0.863–0.957	<0.001	1.91	5.29	23.83	Acceptable
Procrastination in physical health promotion	12.13 (3.90)	4	0.885	0.93	0.930	0.875–0.961	<0.001	1.03	2.85	23.49	Acceptable
Procrastination in quitting high-risk behaviors	7.52 (2.69)	3	0.901	0.91	0.910	0.842–0.949	<0.001	0.80	2.21	29.39	Acceptable
Procrastination in social and mental health	25.97 (7.32)	10	0.890	0.93	0.869	0.770–0.926	<0.001	2.65	7.34	28.26	Acceptable
Procrastination in spiritual health	10.54 (3.03)	4	0.886	0.86	0.859	0.730–0.923	<0.001	1.14	3.16	29.98	Acceptable
total	75.36 (20.72)	29	0.947	0.96	0.944	0.894–0.970	<0.001	4.90	13.57	18.01	Acceptable

a Intra-class Correlation Coefficient, Cronbach's Alpha, Number of Items.

## 4 Discussion

This study was conducted to develop and validate a scale for measuring HRP in nurses. The latest version of this scale consists of 29 items and five factors, including procrastination in maintaining physical health, procrastination in physical health promotion, procrastination in quitting high-risk behaviors, procrastination in social and mental health and procrastination in spiritual health. The World Health Organization (WHO) defines health as “a state of complete physical, mental and social well-being, not just the absence of disease” [23]. In recent decades, the WHO's definition has been refined by adding a fourth dimension, i.e. spiritual health [24]. The dimensions of the scale in the present study are consistent with WHO's definition of health; however, the mental and social dimensions were merged into one. Since many aspects of mental and social health (such as social relationships with colleagues, financial issues, and activities that promote social status) are mutual, this merge seems reasonable.

At the top of the scale, we added the following sentences for all the items: “Without any rational reasons for the delay and despite expecting to be worse off”. Studies have shown that not all delays can be considered procrastination; rather, delays must be without a rational reason and the procrastinator must be aware of the negative consequences of the delay and, in fact, any prudent person must be able to guess the negative consequences of delaying the task [25]. In the same line, a study on procrastination suggests that expecting to be worse off due to the delay is the main component of academic procrastination [26].

The results of the present study showed that the NHRPS has acceptable reliability and validity. All the face and content validity indices obtained had acceptable values. The factor loading of the NHRPS was 0.41–0.98. The factor loading of the Norwegian version of Irrational Procrastination Scale (IPS), was 0.32–0.68 in a sample of students from different universities and 0.48–0.77 in another sample consisting of students from one university [27]. Moreover, the results of the present study showed that, with 29 items and five factors, this scale can explain 55% of the variance in NHRP. A study was conducted to assess the psychometric properties of the General Procrastination Scale. With 23 items and four factors (academic, workplace, medical and civic responsibilities related procrastination), The General Procrastination Scale had a predictive power of 31.25% for changes in procrastination [28]. The Multidimensional Academic Procrastination Scale with 15 items and three factors explained 53% of procrastination [29]. Furthermore, in a psychometric study of the Norwegian IPS, one factor could predict 39.35% of the changes in procrastination in a student sample from different universities and predict 44.72% of the changes in another student sample from one university [27]. These contradictory results could be due to the different procrastination domains examined (academic, general and health), the way the concept of procrastination was defined to generate the items and the different study populations, especially because the nursing community, which was examined in this study, could be a major source of contradiction. The number of NHRP factors in this study was more than the number of procrastination factors in the other domains. As the NHRPS covers a wide range of tasks concerning health, the large number of factors in the scale is more justifiable than the other procrastination domains.

The NHRPS had a Cronbach's alpha of 0.947 overall and the Cronbach's alpha of its dimensions ranged from 0.885 to 0.901. The other procrastination scales showed a good reliability. The Cronbach's alpha for the Short Form of the Academic Procrastination Scale was 0.87 [13]. The Cronbach's alpha was 0.93 for the PPS and 0.85 for the IPS [30]. The Cronbach's alpha for the students' procrastination tendencies was 0.929 [31]. A Cronbach's alpha value more than 0.7 is considered acceptable [32]. Since the Cronbach's alpha of the entire scale and its dimensions were acceptable, the NHRPS items can be said to have an appropriate homogeneity.

The NHRPS also has a good stability, as its total ICC coefficient was 0.944 (p < 0.001) and the ICC for its dimensions ranged from 0.859 to 0.930 (p < 0.001), which is quite comparable with the ICC coefficient of the Spanish version of the IPS, which was 0.84 [33].

In order to verify the goodness of fit of the final factor structure model of NHRPS, the confirmatory factor analysis and Chi-square test were used. The results of Chi-square test were found to be significant, because the Chi-square value is easily influenced by the size of the sample [34], the fit of model was also investigated with respect to other indicators. We estimated several models to take the best fit (one factor, two factors, three factors, four factors five factors and six factors). The current model, namely, the five factors, showed the best fit indices. All indicators confirmed the final fit of model with five factors.

### 4.1 Strengths

One of the advantages of this scale is that it has been constructed based on the definition of the HRP concept and its characteristics in nurses’ experience and perceptions. Also, the response time of the scale is not long and nurses can respond to all the items in just 10 min.

### 4.2 Limitations

One of the limitations of this study was that nurses are more likely than other people to notice the consequences of delaying their health behaviors, and their decisions about procrastinating their health tasks may be different from other people; therefore, one should be careful in generalizing the results of this scale, i.e. it has a limited external validity.

Since procrastination is viewed as a negative attribute, another limitation of this study was the bias of social desirability, which would make the participants try to demonstrate themselves in ways members of the society would better expect. To control this limitation, an emphasis was placed on the anonymity of the questionnaires and all the participants were fully briefed on the importance of honest responding to allow the proper assessment of the scale's validity and reliability. Moreover, a confirmatory factor analysis is necessary to confirm the factor structure of this scale, which was not performed in this research due to the time constraints, and future studies are recommended to carry out a psychometric assessment of the scale via a confirmatory factor analysis.

## 5 Conclusion

The final product of this study was the design of an HRP scale for nurses with five dimensions and 29 items, scored based on a 5-point Likert scale (from ‘never’ to ‘always’), and with good face validity, content validity, construct validity, internal consistency and stability indices. The HRP scale can be used in hospitals and clinics to measure nurses' health related procrastination, research purposes, identifying nurses procrastinating in the health domain and evaluating health interventions and promoting nurses' health. Moreover, in researches that are the basis of health policies, the use of HRP scale will provide vital information to managers and decision makers of the health system.

## Funding statement

This research was supported by 10.13039/100012021Iran University of Medical Sciences with the grant number 1395-03-123-29560.

## Author contribution statement

Mahdi Basirimoghadam; Forough Rafii: Conceived and designed the experiments; Performed the experiments; Analyzed and interpreted the data; Wrote the paper.

Abbas Ebadi: Performed the experiments; Contributed reagents, materials, analysis tools or data; Wrote the paper.

## Data availability statement

Data will be made available on request.

## Declaration of competing interest

The authors declare that they have no known competing financial interests or personal relationships that could have appeared to influence the work reported in this paper.

## References

[1] Vasconcelos S.C., de Souza S.L., Sougey E.B. (2016). Nursing staff members mental's health and factors associated with the work process: an integrative review. Clin. Pract. Epidemiol. Ment. Health.

[2] Haghbin M., Pychyl T.A., Sirois F.M., Pychyl T.A. (2016). Procrastination, Health, and Well-Being.

[3] Perry L., Xu X., Gallagher R., Nicholls R., Sibbritt D., Duffield C. (2018). Lifestyle health behaviors of nurses and midwives: the ‘fit for the future’study. Int. J. Environ. Res. Publ. Health.

[4] Bender B.G. (2014). Can health care organizations improve health behavior and treatment adherence?. Popul. Health Manag..

[5] Kroese F.M., de Ridder D.T. (2016). Health behaviour procrastination: a novel reasoned route towards self-regulatory failure. Health Psychol. Rev..

[6] Rebetez M.M.L., Rochat L., Barsics C., Van der Linden M. (2018). Procrastination as a self-regulation failure: the role of impulsivity and intrusive thoughts. Psychol. Rep..

[7] Svartdal F., Granmo S., Færevaag F.S. (2018). On the behavioral side of procrastination: exploring behavioral delay in real-life settings. Front. Psychol..

[8] Zhang S., Liu P., Feng T. (2019). To do it now or later: the cognitive mechanisms and neural substrates underlying procrastination. WIREs. Cogn. Sci..

[9] Abbasi I.S., Alghamdi N.G. (2015). The prevalence, predictors, causes, treatment, and implications of procrastination behaviors in general, academic, and work setting. Int. J. Psychol. Stud..

[10] Sirois F.M. (2016). Procrastination, Health, and Well-Being.

[11] Doty D.H., Wooldridge B.R., Astakhova M., Fagan M.H., Marinina M.G., Caldas M.P., Tunçalp D. (2020). Passion as an excuse to procrastinate: a cross-cultural examination of the relationships between Obsessive Internet passion and procrastination. Comput. Hum. Behav..

[12] Tuckman B.W. (1991). The development and concurrent validity of the procrastination scale. Educ. Psychol. Meas..

[13] Yockey R.D. (2016). Validation of the short form of the academic procrastination scale. Psychol. Rep..

[14] Svartdal F., Pfuhl G., Nordby K. (2016). On the measurement of procrastination: comparing two scales in six European countries. Front. Psychol..

[15] Lay C.H. (1986). At last, my research article on procrastination. J. Res. Pers..

[16] McCown W., Johnson J., Petzel T. (1989). Procrastination, a principal components analysis. Pers. Indiv. Differ..

[17] Lincoln Y.S., Guba E.G. (1985).

[18] Mousazadeh S., Rakhshan M., Mohammadi F. (2017). Investigation of content and face validity and reliability of sociocultural attitude towards appearance questionnaire-3 (SATAQ-3) among female adolescents, Iran. JAMA Psychiatr..

[19] Kaewkungwal J. (2023). The grammar of science: how “good” is your instrument? Outbreak, surveillance, investigation & response (OSIR). Journal.

[20] Shyu M.L., Huang H.C., Wu M.J., Chang H.J. (2018). Development and validation of the self-awareness of falls in elderly scale among elderly inpatients. Clin. Nurs. Res..

[21] Boltz M., Lee K.H., Shuluk J., Secic M. (2020). Development of the care environment scale-long-term care. Clin. Nurs. Res..

[22] Ebadi A., Froutan R., Malekzadeh J. (2019). The design and psychometric evaluation of the emergency medical services resilience scale (EMSRS). Int. Emerg. Nurs..

[23] World Health Organization (2006). http://www.who.int/governance/eb/who_constitution_en.pdf.

[24] Donev D. (2014). Strengthening the fourth dimension of health the spiritual health. Vox. Medici..

[25] Svartdal F., Nemtcan E. (2022). Past negative consequences of unnecessary delay as a marker of procrastination. Front. Psychol..

[26] Steel P., Klingsieck K.B. (2016). Academic procrastination: psychological antecedents revisited. Aust. Psychol..

[27] Svartdal F. (2017). Measuring procrastination: psychometric properties of the Norwegian versions of the irrational procrastination scale (IPS) and the pure ProcrastinationScale (PPS). Scand. J. Educ. Res..

[28] Lodha P., Sharma A., Dsouza G. (2019). General procrastination scale: development of validity and reliability. Int. J. Med. Publ. Health.

[29] González-Brignardello M.P., Paniagua Á Sánchez-Elvira (2023). Dimensional structure of MAPS-15: validation of the multidimensional academic procrastination scale. Int. J. Environ. Res. Publ. Health.

[30] Kim H., Kim H., Lee W.K., Han S., Carlbring P., Rozental A. (2020). Assessing procrastination in Korean: a study of the translation and validation of the Pure Procrastination Scale and a reexamination of the Irrational Procrastination Scale in a student and community sample, Cogent. Psychol..

[31] González-Geraldo J., Hernández F. (2019). HEAD'19, 5th International Conference on Higher Education Advances.

[32] Lamm K.W., Lamm A.J., Edgar D. (2020). Scale development and validation: methodology and recommendations. J. Int. Agric. Ext. Educ..

[33] Guilera G., Barrios M., Penelo E., Morin C., Steel P., Gómez-Benito J. (2018). Validation of the Spanish version of the irrational procrastination scale (IPS). PLoS One.

[34] Kyriazos T.A. (2018). Applied psychometrics: sample size and sample power considerations in factor analysis (EFA, CFA) and SEM in general. Psychology.

